# Preserving fertility in an unconscious patient with Goodpasture syndrome—medicolegal and ethical aspects

**DOI:** 10.1186/s40560-018-0311-y

**Published:** 2018-07-23

**Authors:** Doreen Stark, Ruth Stiller, Min Xie, Damian Weber, Marco Maggiorini, Matthias Peter Hilty

**Affiliations:** 1 Medical Intensive Care Unit, University Hospital of Zurich, Rämistrasse 100, 8091 Zurich, Switzerland; 2 Department of Reproduction Endocrinology, University Hospital of Zurich, Frauenklinikstrasse 10, 8091 Zurich, Switzerland; 3 Department of Urology, University Hospital of Zurich, Rämistrasse 100, 8091 Zurich, Switzerland

**Keywords:** Goodpasture syndrome, Fertility, Legal objectives, Substituted judgment standard

## Abstract

**Background:**

Every day in the ICU, legal issues arise while treating sedated, unconscious, and legally incapacitated patients. Whenever a life-saving treatment cannot be discussed in a timely manner with an unconscious patient, doctors are required by law to act according to the substituted judgment standard. However, if it is not survival that is at stake, but conservation of reproduction and the potential side effects are significant, the decision-making process becomes much more difficult. Legal issues associated with possible harm to the patient on the one hand and ethical issues with presumable benefit of the intervention on the other hand give rise to difficult decisions.

**Case presentation:**

We present the case of a 24-year-old patient with Goodpasture syndrome. Because of rapid aggravation of kidney function and alveolar hemorrhage—the latter requiring an urgent initiation of mechanical ventilation—therapy with steroids, plasmapheresis, and cyclophosphamide was immediately required. Knowledge of the negative impact on fertility brought up the question about sperm cryopreservation. According to the substituted judgment standard, together with the mother of the patient and based on interdisciplinary evaluation of the situation with specialists from the reproductive endocrinology and urology department, the decision for a testicular sperm extraction in the absence of the possibility to obtain the patient’s informed consent was made. Immediate chemotherapy was initiated and continued after the procedure. The patient recovered from the acute illness and was informed retrospectively about the testicular sperm extraction, which he received extremely positively.

**Conclusion:**

Our aim is to highlight the legal objectives and ethical aspects of a non-lifesaving but fertility-preserving intervention in an unconscious patient. The need for decision-making in this kind of situation is rare and therefore challenging. The present case may serve to encourage and guide other doctors in similar situations.

## Background

**Fig. 1:**
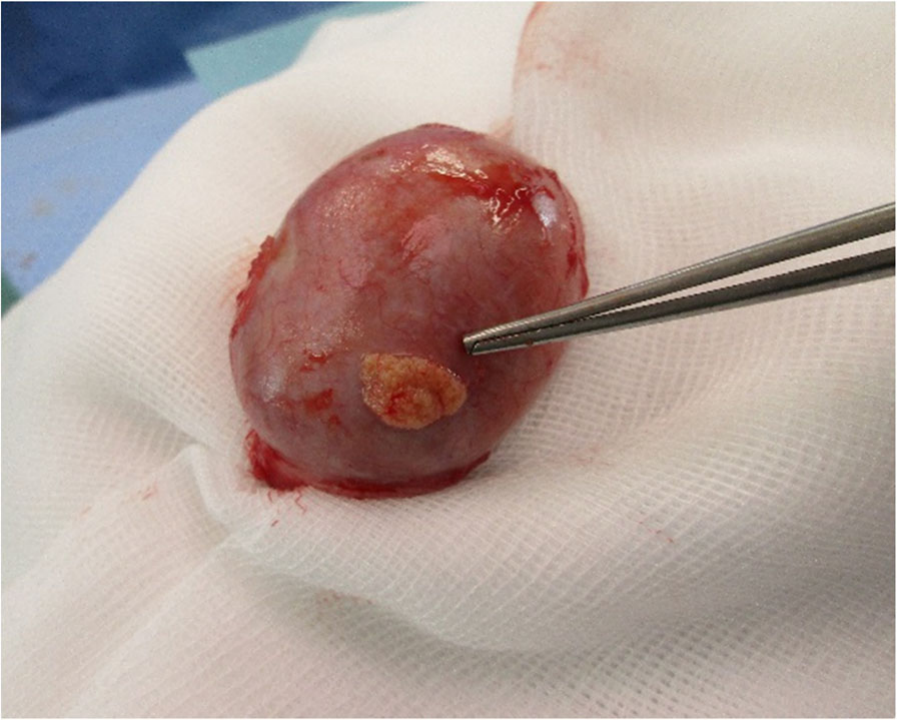
Testicular sperm extraction in left testis. Incision of tunica albuginea and pulpa of testis can be removed with scissors

**Fig. 2:**
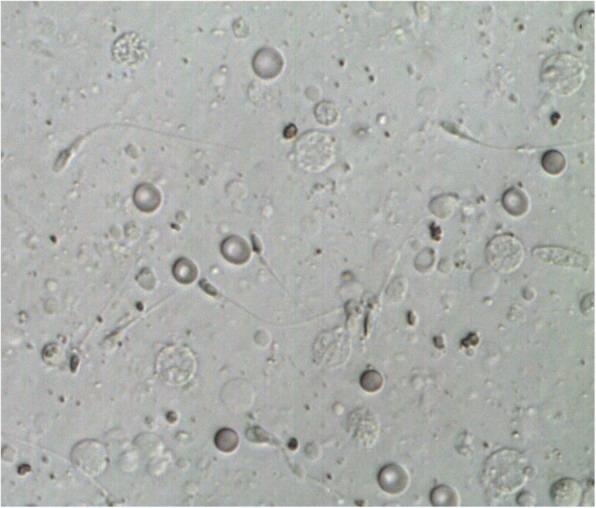
Microscopic images of testicular sperm extraction sample before cryopreservation

It is well established that many benign or malignant diseases by themselves or as a result of treatment impair male fertility. Besides, treatment for autoimmune disorders such as Goodpasture syndrome (GS) is also interfering with the reproductive system. GS is an anti-glomerular basement membrane antibody (anti-GBM) disease in which circulating antibodies are directed against an antigen intrinsic to the glomerular basement membrane, thereby resulting in acute or rapidly progressive glomerulonephritis and/or pulmonary hemorrhage. Like other autoimmune conditions, the anti-GBM disease is thought to result from an environmental insult in a person with genetic susceptibility. An initial insult to the pulmonary vasculature is required for exposure of the alveolar capillaries to the anti-GBM antibodies. Environmental factors that may lead to such exposure include the following: exposure to organic solvents or hydrocarbons, smoking, infection (e.g., influenza A2), cocaine inhalation, and exposure to metal dust [1–4]. Symptoms like malaise, chills, fever, or arthralgia may precede or develop concurrently with pulmonary or renal manifestations. When the disease affects the lungs, it may come to hemoptysis and/or other pulmonary symptoms like cough, dyspnea, and shortness of breath. Massive pulmonary hemorrhage leading to respiratory failure may occur. Renal manifestations include hematuria, edema, high blood pressure, and eventually uremia. The diagnosis of anti-GBM disease is established in older children as well as in adults by the presence of pulmonary hemorrhage, positive anti-GBM antibodies, or after kidney biopsy. Treatment of choice with plasmapheresis combined with prednisone and a cytostatic like cyclophosphamide [1–3, 5, 6] should be initiated as soon as possible. Knowing that treatments containing high doses of alkylating agents (such as cyclophosphamide) present the highest level of risk for gonadal impact (azoospermia with irreversible mutagenic effect on all stages of spermatogenesis), patients should be counseled on fertility preservation prior to treatment [7–9]. In males, the established method to secure fertility is cryopreservation of sperm, which can later be used for assisted reproduction techniques [8]. If patients are unable of producing a semen sample with ejaculation, the most common methods for sperm retrieval include fine-needle aspiration, testicular sperm extraction (TESE), and micro-TESE (microsurgical TESE using magnification) [9]. TESE involves an incision of the tunica albuginea and retrieval of seminiferous tubules (Figs. 1 and 2).

Sperm retrieval rates with TESE are significantly higher than that with aspiration [10–12]. Microsurgical skills are not necessary, and the procedure can be performed quickly in local anesthesia in the surgeon’s office or even in an intensive care unit [13]. Using TESE and micro-TESE, complications such as inflammation, infection, or local hematoma with impaired testicular blood flow or even complete devascularization of the testes are possible [14]. Documentation of complication rates is rare in literature and only includes small sample size studies. The rates vary from 2% for wound infection and 2.5% for ischemic atrophy of the testis to 51% and 82% for ultrasonographic hematoma and unspecific signs of inflammation 1 month after the procedure [14, 15]. Consulting the recommendations of the American Society of Clinical Oncology on fertility preservation in cancer patients of 2013, the possibility of infertility and options for fertility preservation should be discussed with all patients who are to be treated in their reproductive years [7]. Therefore, we considered preserving fertility using TESE and sperm cryopreservation in a young unconscious male patient prior to chemotherapy for an autoimmune disease. We discuss medico legal and ethical aspects.

## Case presentation

The 24-year-old, adipose (BMI 41, 9 kg/m^2^) male patient had a 2-week history of bloody sputum accompanied by progressive dyspnea, urine of light pink color, and fever up to 39 °C. Because of a long duration car travel to Serbia and Montenegro prior to his complaints, a lung CT scan in the emergency department excluded pulmonary embolism. However, bilateral ground glass opacities and bihilar lymphadenopathy were documented—findings that were new as compared to a CT scan 2 years prior, which had been performed after suspected trauma. Together with the changes in the lungs and an elevated CRP of 47 mg/l as well as leukocytosis of 17.3 G/l, an empirical antibiotic regime with ceftriaxone and levofloxacin was started. Because of an increasing oxygen demand, he was admitted to the intensive care unit (ICU). On the ICU, oxygenation deteriorated rapidly under non-invasive ventilation so that the patient had to be immediately intubated with mechanical ventilatory support.

In addition, an acute impairment of kidney function with a calculated glomerular filtration rate of 42 ml/min was present, suggesting an autoimmune process with kidney and pulmonary involvement. Laboratory analysis showed positive results for anti-GBM with a high titer of 151 E/ml, and diagnosis of Goodpasture syndrome was made. Other autoimmune antibodies (ANCA) were negative. Besides, at this point, the mother stated of having a GS herself with kidney transplantation several years ago. Because of rapid aggravation of kidney function and alveolar hemorrhage, therapy with steroids, plasmapheresis, and cyclophosphamide was immediately required. Knowledge of the negative impact on fertility brought up the question about sperm cryopreservation. Assessment of the patient’s will with consultation of the patient’s mother revealed that he presumably would wish to reproduce in the future, even though there were no concrete plans known to the mother at the moment. The situation was discussed in consultation with specialists from the reproductive endocrinology and urology department, leading to the interdisciplinary decision for a testicular sperm extraction in the absence of the possibility to obtain the patient’s informed consent. The procedure took place on the ICU during nighttime in order to avoid a delay in life-saving treatment. Immediate chemotherapy was initiated and continued according to the CYCLOPS study protocol [16]. Alongside a step down regimen of corticosteroids adapted to anti-GBM value as well as daily plasmapheresis for a total of 14 days took place. Following initial therapy, acute kidney failure developed with need of continuous hemodialysis. A septic shock following a non-occlusive mesenteric ischemia required a specific antibiotic therapy as well as terminal ileum resection with ileostomy, subtotal colectomy, and Hartmann procedure. Because of a severe metabolic-toxic and possibly medication-induced delirium, weaning from mechanical ventilatory support failed and a dilative tracheotomy was performed. Finally, the patient recovered from the acute illness with successful removal of the tracheal cannula but ongoing need for renal replacement therapy. Regularly, he is scheduled for an intermittent hemodialysis. For control of GS, he receives a low dosage of steroids (prednisone 5 mg/day) together with close monitoring of anti-GBM antibodies (actually 14 E/ml). After recovery, the patient was informed about the interventions (especially TESE) performed during his stay on the ICU, which he received extremely positively. Informed consent for further storage of the sperms was obtained from the patient at this point.

## Discussion and conclusion

With advances in medicine, especially in treatment for autoimmune and cancer disease, patients face greater longevity and quality of life factors including preservation of fertility and paternity have become significant issues with 76% of childless cancer survivors desiring to have children in the future [17, 18]. From a Japanese study of patients undergoing semen cryopreservation before cancer therapy, we also know that for 80% of these patients, the knowledge about cryopreservation helped them in the emotional battle against the disease [19]. Up to two thirds of patients are azoospermic after chemotherapy [20]. The chance of recovery of spermatogenesis may be affected by the chemotherapeutic regimen and baseline reproductive function of the patient. Notably, alkylating agents, including cyclophosphamide, seem to have the most profound reproductive effects [9, 21]. Adequate sperm will return to the ejaculate sufficient for natural conception in some patients; however, these numbers are poorly defined and fertility preservation options are usually discussed with the patient prior to chemotherapeutic treatment [7]. If promptly referred to a fertility specialist, there is also likely to be little to no significant delay in the initiation of treatment. In an ICU setting, where time for decisions for therapies of life-threatening diseases is short and diagnosis might not be known, while the patient is conscious, the doctors together with relatives have to decide for the patient. This decision is difficult when facing an intervention that is not life-saving but fertility-preserving and complications might occur. It is the substituted judgment standard (the decision that ought to be made for the incapacitated patient, he or she would have made in this situation) that one has to follow. It is not always clear what the patient “would have done” in this situation, and it is interfering with the patients right to self-determination [22].

Civil right in most states, among them Switzerland (Swiss Civil Code art. 378, [23]), ranks persons who may represent a patient if he or she is unconscious. The first in line would be the person documented in a patient provision or a legal advisor, followed by spouse, people who live together with the patient in one household, and finally, descendants or parents, if they are in regular contact with the patient. This course of action with ranking the persons who may represent the patient’s will applies in Swiss legislation to all medical interventions that are considered in legally incapacitated patients, not only in life-saving procedures. In the present case, according to the substituted judgment standard as represented by the mother of the patient who was to our knowledge the closest person to the patient as stated above by civil right, it was clear that the patient would like to reproduce in the future. Therefore, the non-lifesaving but fertility-preserving procedure was carried out.

In most ethical considerations, reproductive liberty is regarded as an important basic human right, and therefore, if fertility is threatened, an individual should be able to take measures to preserve it. However, it is a liberty right, something an individual can choose to pursue but not something that society is required to provide. Other considerations are the ethical principles of non-maleficence, beneficence, and justice. According to the concept of “do no harm,” doctors have a duty to prevent damage or repair damage caused by medical treatment, e.g., chemotherapy [24]. Thus, an interdisciplinary risk-benefit evaluation of the procedure was performed. Considering the low risk of the intervention even in a cardiopulmonary instable situation, the decision was made in favor of the procedure and fertility preservation. On the other hand, there might be concerns that fertility preservation may delay life-saving treatment, but as mentioned above, sperm banking rarely delays treatment. In the case presented, an experienced urologist performed TESE within 90 min of admission to the ICU in analgosedation during nighttime and life-saving therapy was started immediately afterwards. An embryologist also took the samples immediately to the IVF laboratory and conducted cryopreservation after viable spermatozoa were found in the biopsied samples. It may also be argued that fertility preservation is not in the best interest of the future child because of fear that the child might inherit the disease as well. Although some autoimmune disorders have a hereditary component, it is not known for GS, but there might be an increased disposition to develop the disease rather than inheriting the disease itself. Maybe the patient also had a genetic susceptibility, with his mother having GS. The occasional, although not recently, consumption of cocaine might have triggered the disease in this patient [25]. Further, the disease is treatable and relapses are rare. Therefore, it is ethically not correct to deny a patient with GS for his fertility.

Untreated patients do not recover renal function and have substantial mortality, particularly from pulmonary hemorrhage. The introduction of oral immunosuppression did not obviously alter the prognosis of the disease. However, the use of plasma exchange in combination with prednisolone and cyclophosphamide dramatically improved outcomes [3, 5, 26], suppresses antibody production, and may preserve renal function [2]. Therefore, the decision for full therapy was made in this young patient.

Although in Switzerland all people have a compulsory insurance that covers most of medical expenses, the moderate cost associated with the procedure described and the cryopreservation usually are exempted. Cost might thus also be an argument pro or con the procedure, depending on the financial status of the patient. If medical insurance would cover these expenses, the discussion about legal issues of this procedure will probably still be the same. However, the patient’s legal representative might feel more at ease to decide for the procedure and cryopreservation if it would not result in a financial burden to the patient.

In conclusion, the present case highlights the importance of considering medical, legal, and ethical aspects when making decisions on ICU in unconscious patients. These decisions may go beyond the question of immediately life-saving interventions but have to be balanced carefully with potential adverse effects and based on interdisciplinary consent. In the presented case, an invasive procedure was applied in accordance with the patient’s substituted judgment standard and local legislation in order to preserve the young patient’s fertility.
